# Diagnostic performance of a single breath-hold lung MRI scan with AI-powered compressed sensing for nodule detection in comparison to photon counting detector-CT

**DOI:** 10.1007/s00330-026-12738-7

**Published:** 2026-07-10

**Authors:** Anna Palmisano, Giulia Piccinni, Davide Serra, Elisa Bruno, Giulio Ferrazzi, Davide Vignale, Carlo Tacchetti, Antonio Esposito

**Affiliations:** 1 Advanced Imaging for Personalized Medicine Unit, Experimental Imaging Center, IRCCS San Raffaele Hospital, Milan, Italy; 2 Vita-Salute San Raffaele University, Milan, Italy; 3 S-RACE (San-Raffaele Ai CEnter), Vita-Salute San Raffaele University, Milan, Italy; 4 Philips Healthcare, Milan, Italy

**Keywords:** MRI, Lung, Screening, Computed tomography

## Abstract

**Objective:**

The aim was to evaluate the performance of a single-breath-hold accelerated 3D-T1w-FFE 3-T MRI sequence for screening lung nodules, compared with photon-counting detector CT (PCD-CT), which served as the reference standard.

**Materials and methods:**

In this single-center prospective study, 148 healthy adults underwent lung 3-T MRI between June 2024 and June 2025 using a single-breath-hold, AI-aided compressed sensing (AI-CS)- accelerated 3D-T1-FFE sequence (acceleration factor of 9) to evaluate for the presence of lung nodules. Patients scanned within 24 h using PCD-CT, for comparison, were enrolled. Lung-RADS scores were evaluated per patient, and all nodules were assessed for size and composition. The diagnostic performance of MRI was compared with PCD-CT, including interobserver and intermodality agreement, using Lin’s Concordance Correlation Coefficient (CCC).

**Results:**

97 patients were enrolled (65 men, 67%; mean age 58 ± 12 years old) and 33 (34%) were active smokers. MRI and CT showed complete agreement in the Lung-RADS 4A and 4B categories. MRI demonstrated an overall sensitivity of 83.1% (95% CI: 73.2–89.9) for solid nodules, increasing to 98.1% (95% CI: 90.2–99.7) for nodules ≥ 4 mm. Lin’s CCC for nodule size was 0.985, indicating excellent agreement, with MRI showing a slight underestimation of 1.02 mm (95% CI: [−1.16, 3.20]) in Bland–Altman analysis. MRI failed to detect calcified nodules < 4 mm, which accounted for 51.2% of undetected nodules.

**Conclusions:**

A single breath-hold AI-CS accelerated 3D-T1-FFE MRI scan can be a valuable tool for solid lung nodule screening, showing very good agreement with CT.

**Key Points:**

***Question*** *May lung MRI using an optimized AI-CS accelerated 3D-T1-FFE sequence acquired in a single breath hold be a radiation-free alternative to CT in lung cancer screening?*

***Findings*** *Lung MRI using AI-CS 3D-T1-FFE sequence achieved an overall accuracy of 87.3% for detecting solid nodules overall, increasing to 97.7% for nodules ≥ 4 mm.*

***Clinical relevance*** *Lung MRI using an optimized AI-CS 3D-T1-FFE is a promising alternative to chest CT in lung nodules screening, offering high diagnostic accuracy, especially for clinically relevant nodules, excellent Lung-RADS classification agreement, and short acquisition time (17.2 s [IQR, 17.2–17.3])*.

**Graphical Abstract:**

**Figure:**
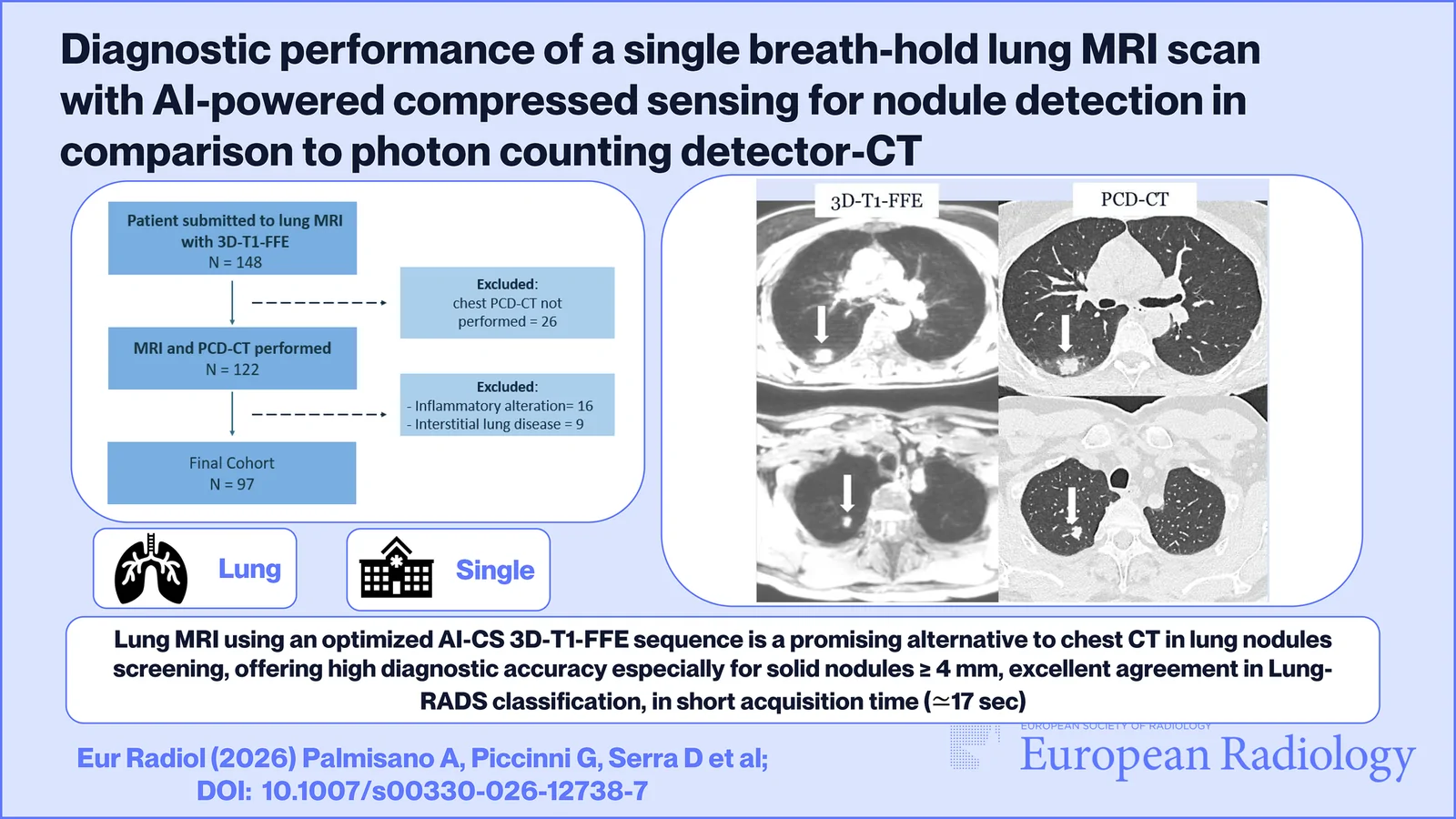

## Introduction

Primary lung cancer is the second most common malignancy and the leading cause of cancer-related death worldwide [1].

Computed tomography (CT) is the standard imaging modality for detecting pulmonary nodules due to its availability, excellent contrast and spatial resolution, and rapid acquisition [2].

Low-dose CT (LDCT) screening in high-risk patients has shown a 20% reduction in cancer-related mortality [3, 4], through early-stage detection [5], but requires repeated scans [6], exposing patients to cumulative radiation, with a lifetime risk increase of radiation-induced lung cancer by 0.5%–5.5% [7].

Magnetic resonance imaging (MRI) offers radiation-free imaging and superior soft-tissue contrast, but lung imaging remains challenging due to low proton density, extremely short T2*, susceptibility artifacts at air-tissue interfaces, and cardiac and respiratory motions [8].

Recent advances in MRI hardware and software have improved image quality, prompting the Fleischner Society to consider MRI a valuable radiation-free alternative for lung cancer screening [9]. Additionally, Markov modeling demonstrated that MRI could offer similar life expectancy benefits and potentially better cost-effectiveness than CT [10].

The main barrier to clinical implementation of lung MRI is scan duration. Scanning time of several minutes entails patient compliance issues, increased costs and infrastructure requirements that disrupt screening workflow [11].

Artificial intelligence-assisted compressed sensing (AI-CS) enables faster scans by combining incoherent k-space sampling with deep learning-based reconstruction and denoising. This approach, primarily applied in brain and musculoskeletal imaging, has facilitated faster acquisition protocols, reduced patient discomfort, and improved diagnostic capabilities [12, 13].

AI-CS has shown promise in lung screening by reducing scan time while maintaining image quality and diagnostic accuracy [14]. In a prior study by Ziegelmayer et al [14], although high isotropic spatial resolution (1 mm) was achieved using an AI-accelerated 3D gradient echo sequence, the total scan time remained several minutes, preventing single breath-hold acquisition—a key requirement for rapid screening.

Shorter acquisition times reduce motion artifacts, improve patient compliance, and better simulate the fast workflow of LDCT. Therefore, the objective of this study is to assess the diagnostic sensitivity of a lower-resolution (2 mm isotropic) AI-CS 3D-T1-FFE 3-T MRI protocol for detecting pulmonary nodules, entirely acquired during a single breath-hold, using photon-counting detector CT (PCD-CT) as the standard of reference.

## Materials and methods

### Study design

This is a single-center prospective study approved by the local Ethics Committee (AIMOMICS-BANK CET 233-2024); written informed consent was obtained.

We screened consecutive asymptomatic adult patients (aged ≥ 18 years) who underwent lung 3-T MRI, as part of whole-body MRI screening, between June 2024 and June 2025.

Inclusion required a same-day non-contrast chest CT on PCD-CT, used as the reference standard. Exclusion criteria were: (i) refusal to provide consent; (ii) pediatric patients (age < 18 years); (iii) chest CT not performed; (iv) interstitial lung disease or inflammatory alterations.

**Fig. 1:**
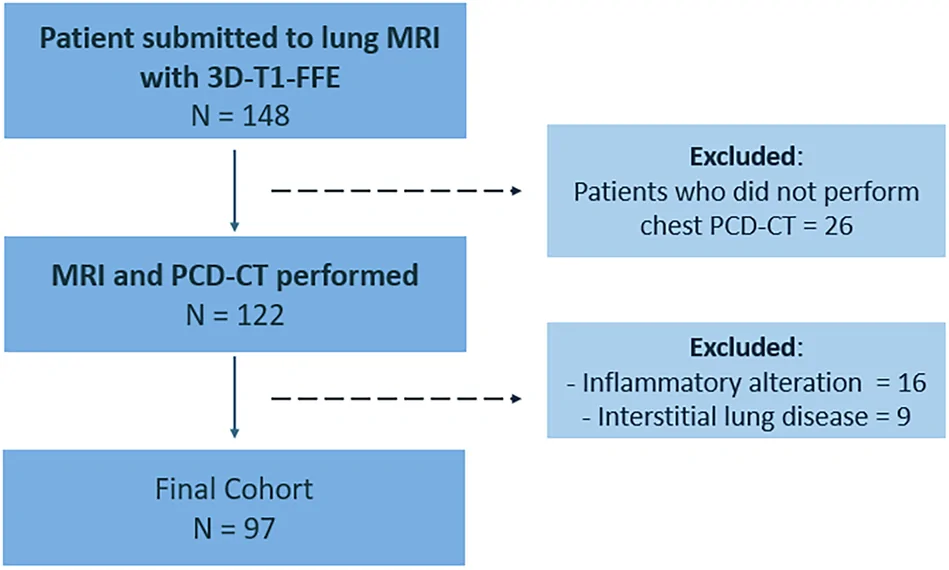
Enrollment flow-chart

The enrollment flow-chart is shown in Fig. 1.

Clinical data collected included demographics (sex and age) and lung cancer risk factors (smoking, family or personal history of lung disease, occupational exposure).

### Lung MRI and chest CT acquisition protocol

MRI was performed on a 3-T system (Philips 7700, Philips Healthcare) equipped with a 32-channel cardiac receiver coil and a gradient system capable of a maximum amplitude of 65 mT/m and a slew rate of 220 T/m/s. Chest MRI was performed with the patient in the supine position, without contrast agent administration, using a 3D T1-weighted fast field echo (FFE) Dixon Cartesian coronal scan, acquired with a 2 × 2 × 2 mm isotropic spatial resolution. To minimize echo time and improve visualization of pulmonary nodules, especially of the first echo, a hard, non-selective transmit pulse was used. Moreover, the default manufacturer oversampling factor of 2 along the foot-head (FH) readout direction was applied to prevent signal aliasing. The sequence employed 2 echoes (TE1/TE2 = 0.9/2.0 ms) and a repetition time (TR) of 3.1 ms, flip angle (FA) of 10°, water-fat shift of 0.35 pixels, and a field of view of 380 × 442 × 300 mm³. The oversampling factor in the right–left (RL) direction was individually adjusted based on each patient’s chest size, resulting in a median breath-hold duration of 17.2 s [IQR 17.2–17.3], with an oversampling margin of up to 180 mm on either side.

Image reconstruction was performed using an AI-CS framework (SmartSpeed, Philips Healthcare) with an acceleration factor of 9. This technology employs a deep learning-based reconstruction algorithm, the Adaptive-CS-Net, which is integrated into the iterative reconstruction process [15]. We used a denoising level set to 40% to obtain a balance between noise reduction and structural details preservation.

A chest CT study was performed on a PCD-CT system (NAEOTOM Alpha; Siemens Healthineers) during end-inspiration breath-hold, without contrast media, using a low dose protocol (IQ level 15, Sn 100 KV, slice thickness 0.8 mm, increment 0.6 mm, matrix 1024^2^, BI56, QIR3).

Image reconstruction was performed in axial and coronal views.

The median dose-length product (DLP) was 34.9 [IQR, 34.5–57] mGy*cm, while the median volumetric CT dose index (CTDIvol) was 0.9 [IQR 0.7–1.1] mGy.

### Image analysis

Images were reviewed anonymously by two board-certified radiologists with more than 15 years of experience in thoracic radiology on commercially available picture archiving and communication systems, in consensus.

Standardized window settings were applied for CT (level, –550 HU; width, 1600 HU) and MRI (level 110 SI; width 700 SI).

All detected nodules were analyzed on a per-nodule basis for detection performance, including location, size and morphology for both PCD-CT and MRI.

They also assigned Lung-RADS categorization on a per-patient basis: in patients with more than 1 nodule, the one determining the highest category was used for the final Lung-RADS classification, in accordance with standard practice [16].

Lesion size was manually measured as a single diameter for round nodules or as the longest axial diameter for oval lesions.

A second reader with 5 years of experience analyzed the images to assess interobserver agreement.

On PCD-CT, nodules were classified into ground glass opacity (GGO), partially solid nodules, solid nodules, and calcific nodules according to the Fleischner Society Guidelines [17].

### Statistical analysis

Nodule characteristics were summarized using descriptive statistics.

Normality was assessed with the Shapiro–Wilk test. Normally distributed variables are reported as mean ± standard deviation (SD), non-normally distributed variables as median and interquartile range (IQR), and categorical variables as counts and percentages. Comparisons between groups were performed using the Student’s t-test or the Mann–Whitney *U*-test for continuous variables, and the chi-square test or Fisher’s exact test for categorical variables. Odds ratios (OR) with 95% confidence intervals (CI) were calculated for detection rates. Statistical significance was defined as a two-sided p-value < 0.05.

MRI nodule detection performance was evaluated against the CT reference standard, considering composition and size [18]. Detection agreement was assessed using Cohen’s kappa coefficient (*κ*), and paired detection differences were calculated via the McNemar test.

For nodules visible on both modalities, Lin’s Concordance Correlation Coefficient (CCC) was calculated to quantify the degree of agreement in diameter measurements between CT and MRI, taking into account both correlation and deviation from the identity line. Additionally, scatter plots and Bland–Altman plots evaluated systematic or proportional biases in size estimation.

A logistic regression analysis evaluated the influence of nodule characteristics on the probability of MRI detection. In this model, MRI detection (yes/no) was used as the outcome variable, and nodule diameter (measured on CT) and composition (solid vs. calcific) were included as predictors. OR with 95% confidence intervals were estimated to quantify the effect of each variable.

Statistical analyses were performed using Python 3.10, with the support of packages including pandas, statsmodels, pingouin, and matplotlib.

## Results

The study enrolled 97 patients (men 65/97, 67%, mean age 58 ± 12 years). Among them, 33 (34%) were active smokers, 14 (14%) were former smokers, and 1 (1%) had a history of occupational inhalation exposure.

Image quality was good in all lung MRI exams, with a Likert score always ≥ 4 (score 4 in 34 cases; score 5 in 63 cases). The 34 cases with a Likert score of 4 showed mild artifacts due to either respiratory motion (*n* = 10) or cardiac motion (*n* = 24).

Lung MRI detected nodules in 48 patients, while PCD-CT in 63 patients, regardless of composition.

**Fig. 2:**
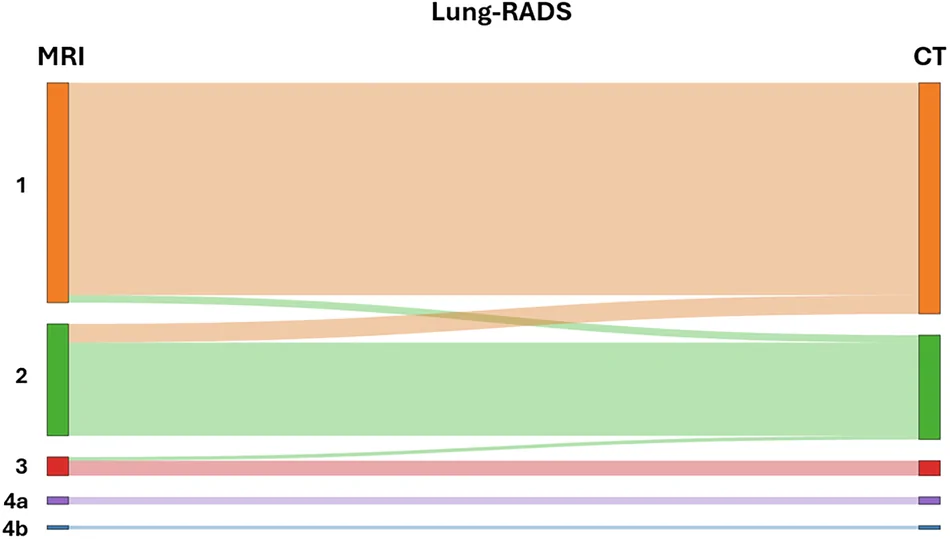
LUNG-RADS classification with MRI and PCD-CT

**Fig. 3:**
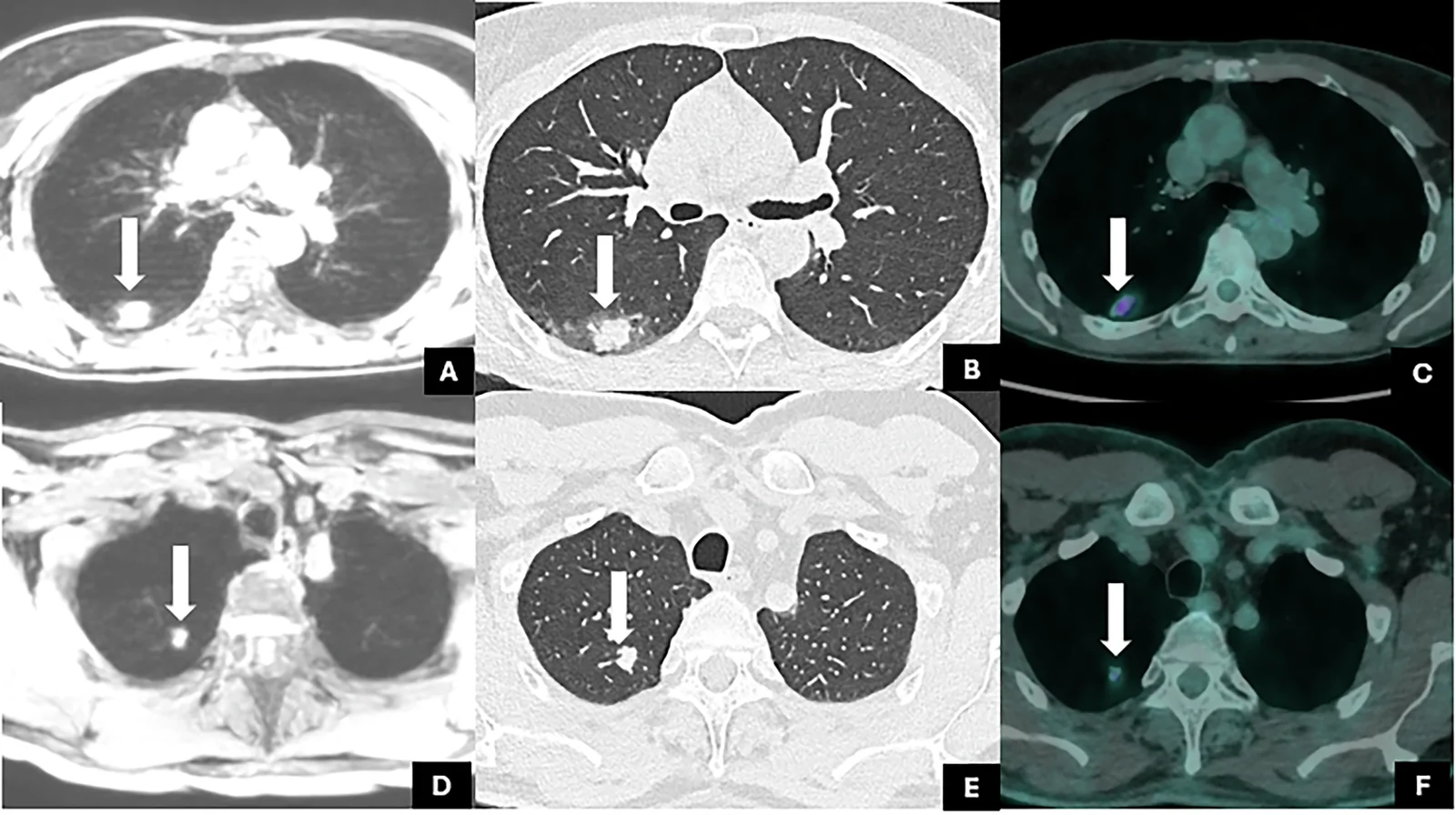
Top images (**A**–**C**): a 57-year-old woman, an active smoker, with a part-solid nodule in the superior segment of the right inferior lobe on 3D-T1-FFE (**A**), considered likely malignant (Lung-RADS 4B). This was confirmed on PCD-CT (**B**) and on FDG-PET (**C**), showing avid uptake in the central solid part. It was diagnosed with invasive adenocarcinoma with predominantly lepidic growth. Bottom images (**D**–**F**): a 56-year-old man, without risk factors, 3D-T1-FFE showed a solid nodule with lobulated edges classified as LUNG-rads 4a (**D**) with a slight underestimation in sizing by MRI (**D**) compared to CT (**E**) (11 mm vs. 12 mm). Pathological uptake was detected by FDG-PET, and histology documented an invasive mixed adenocarcinoma, 80% mucinous and 20% papillary

**Fig. 4:**
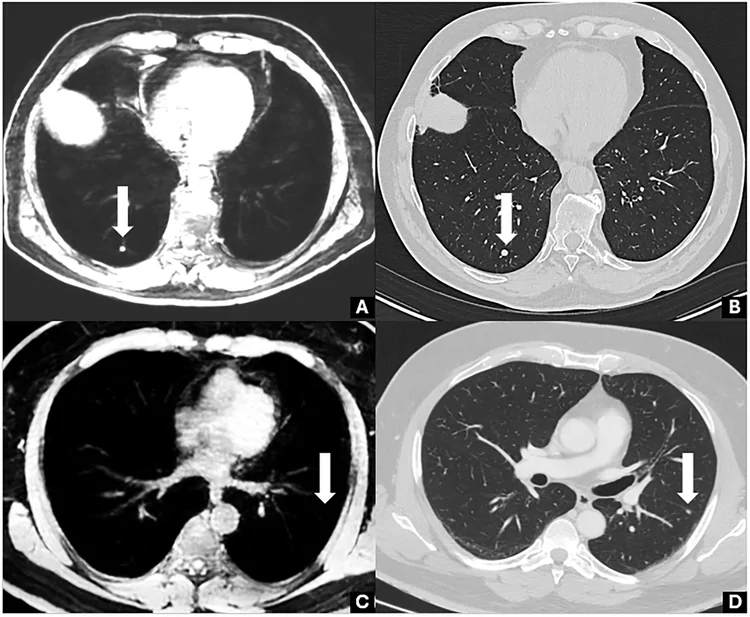
Top: an illustrative case of agreement between 3D-T1-FFE (**A**) and PCD-CT (**B**) in detecting a solid nodule with a long-axis diameter of 6 mm in the lower right lobe. Bottom: an illustrative case of intermodality disagreement, showing a solid intrascissural nodule with a long-axis diameter of 3 mm in the left lower lobe (**D**), which was not detected by MRI (**C**)

**Fig. 5:**
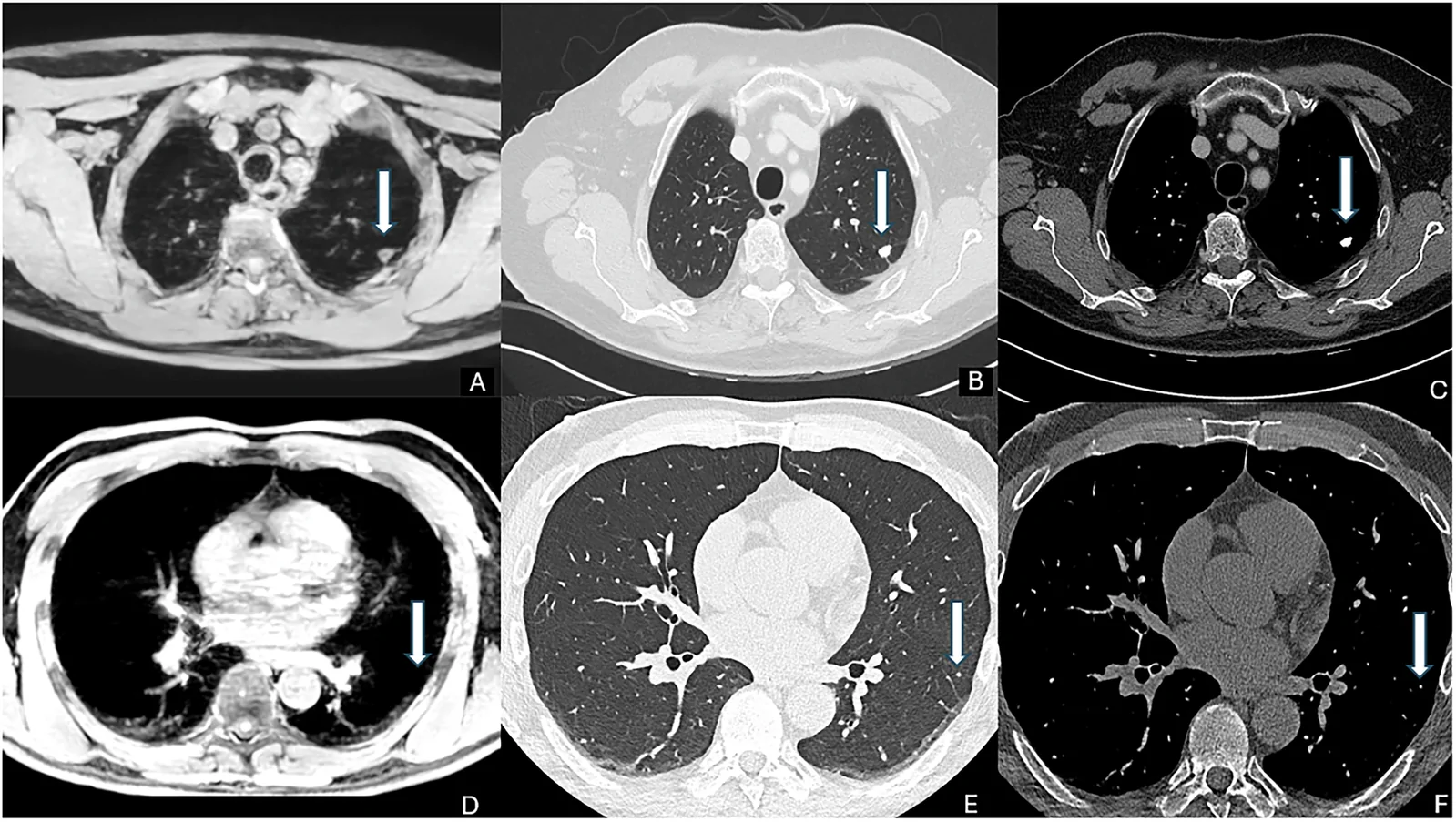
Calcified nodules at lung MRI. 3D-T1-FFE (**A**) shows a 10 mm nodule with thin pleural striae, confirmed as calcified at PCD-CT (**B**, **C**) with matching size. On the bottom, 3D-T1-FFE (**D**) failed in the identification of a small, calcified nodule (2 mm diameter) documented at PCD-CT (**E**, **F**)

MRI and PCD-CT showed strong agreement in Lung-RADS classification (Fig. 2 and Table 1). Specifically, MRI correctly classified suspicious nodules (Table 1 and Fig. 3), but missed 41/118 nodules, resulting in a change from Lung-RADS MRI score 1 to CT score 2 in 2 patients (Figs. 2 and 4). Additionally, 5 patients with large and calcified nodules appeared solid on MRI and were misclassified as Lung-RADS MRI score 2, while they were Lung-RADS CT score 1. One patient classified as Lung-RADS MRI score 3 was Lung-RADS CT score 2 (Figs. 2 and 5).

**Table 1:**
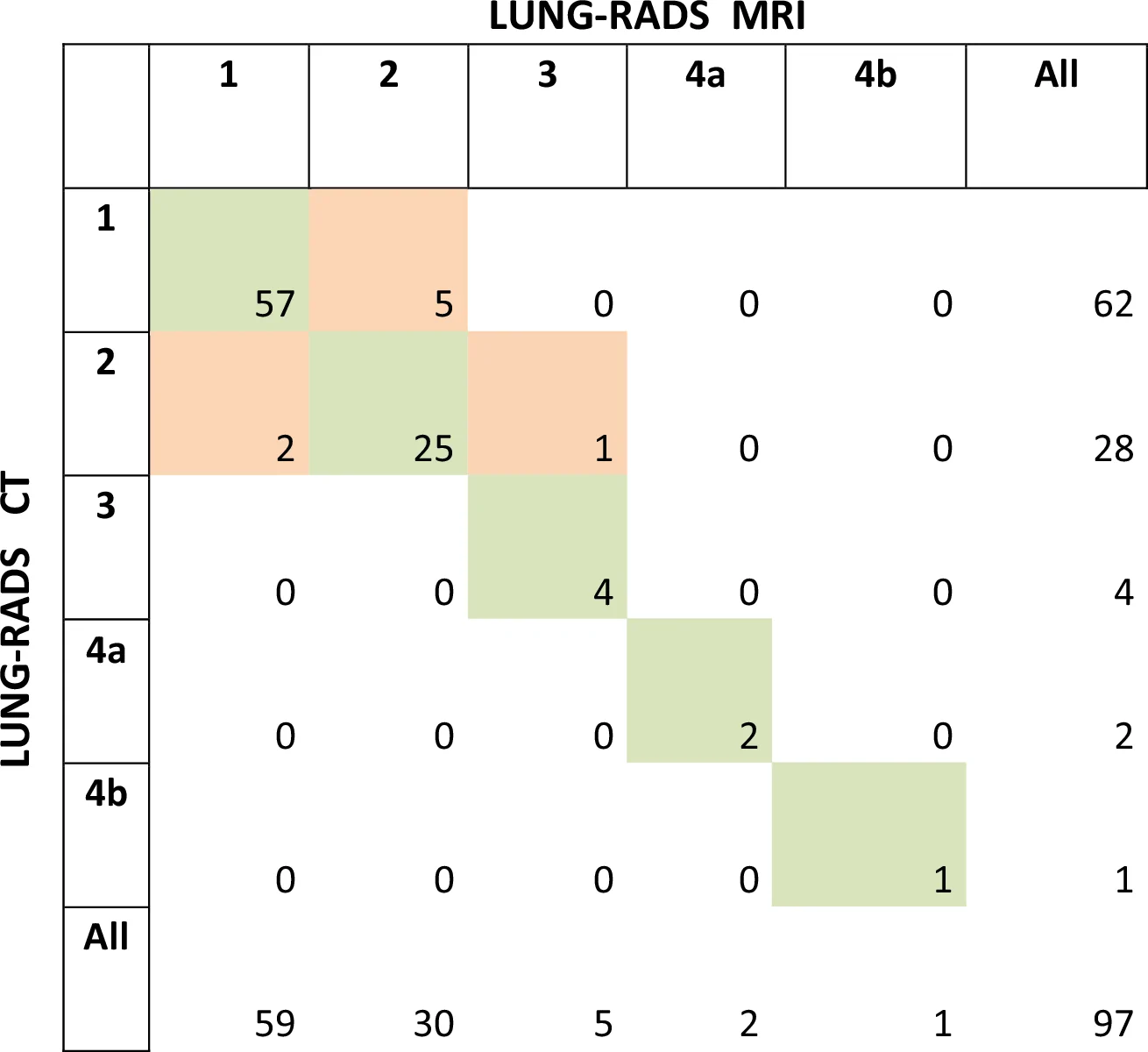
Confusion matrix of Lung-RADS in PCD-CT and lung MRI

Heat map showing agreement between LUNG-RADS TC and LUNG-RADS RM categories. Green cells along the diagonal represent complete agreement between the two assessments, whereas red cells represent disagreement (discordant classifications). The numbers within the cells indicate the frequency of cases for each category combination.

### Overall nodules detection

PCD-CT identified a total of 118 pulmonary nodules with a median size of 4.5 mm [IQR, 3–6] mm, including 73 solid, 4 part-solid, 6 GGO, 23 calcific and 12 part-calcific nodules.

In total, MRI detected 78 nodules, all of which were confirmed by CT, except for one, resulting in a false positive due to an image artifact.

MRI sensitivity varied according to nodule composition: 63/73 solid nodules (88%), 8/12 part-calcific (67%), 2/6 GGO (33%), 1/4 part-solid (25%), and only 2/23 calcific nodules (9%).

**Fig. 6:**
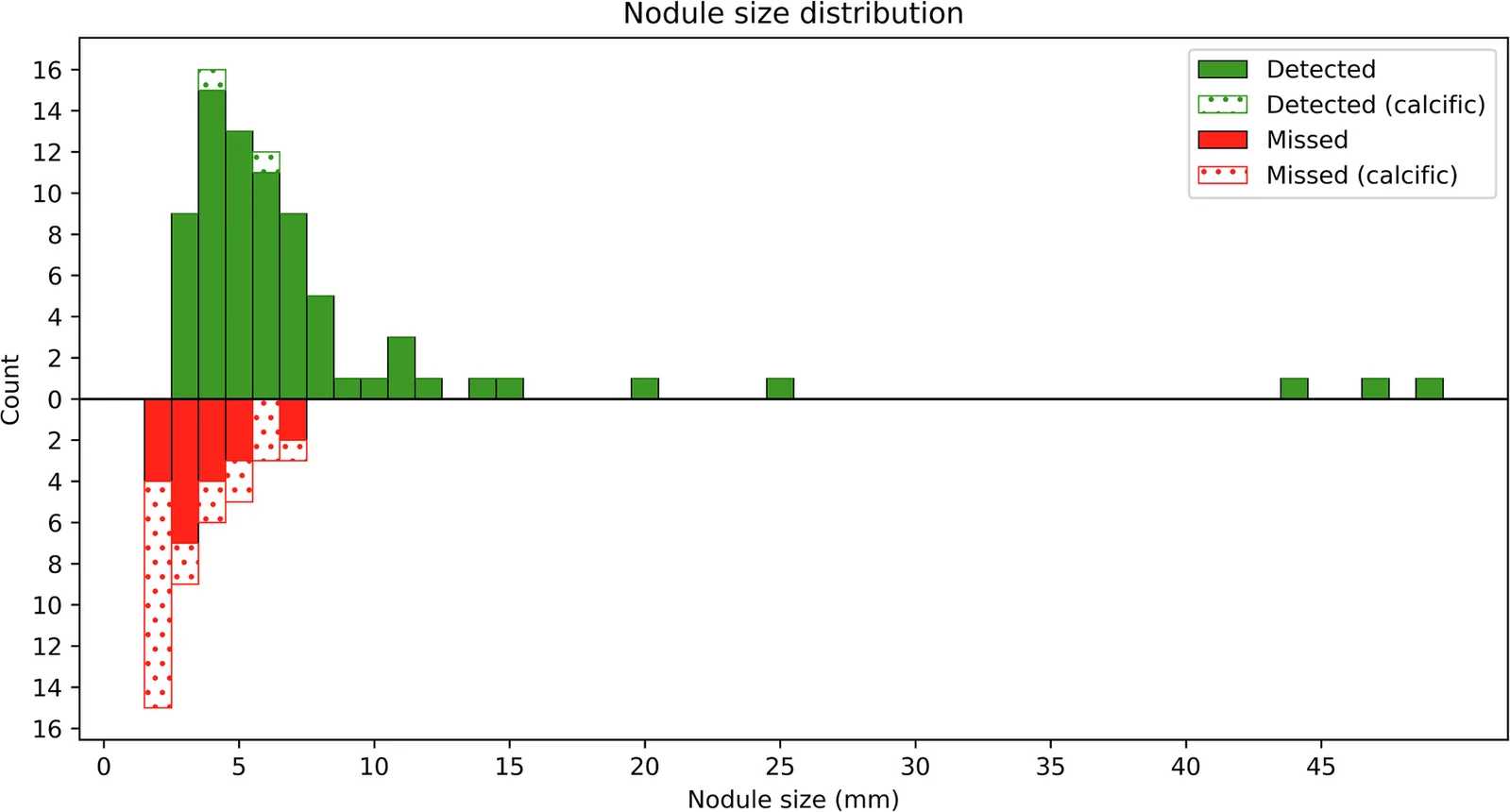
Size distribution plot showing missed and detected nodules.

MRI missed 41 nodules, which had a significantly smaller median diameter on PCD-CT compared to detected nodules (3.0 mm [IQR 2.0–4.5 mm] vs. 4.0 mm [IQR 3.5–7.0 mm]; *p* < 0.001). More than half of the missed lesions were calcific (21/41, 51.2%; median diameter 2.0 mm [IQR 1.5–5.0 mm]), while the remaining 20 (48.8%) were mostly solid, with a median diameter of 2.5 mm [IQR 2.1–3.0 mm] (Fig. 6).

In the subgroup of 77 nodules detected by both modalities, the maximum diameters measured on PCD-CT were significantly larger than those measured on MRI (5.5 mm [IQR, 4.0–7.0 mm] vs. 4.0 mm [IQR, 3.0–6.0 mm]; *p* < 0.001), suggesting a slight systematic underestimation by MRI. Bland–Altman analysis revealed a mean bias of 1.02 mm (95% CI: [−1.16, 3.20] mm) and a CCC of 0.985.

Using CT as the reference standard, MRI achieved a specificity of 97.0% (95% CI: 84.7–99.5) and a positive predictive value (PPV) of 98.7% (95% CI: 93.1–99.8), indicating that nearly all nodules detected by MRI were true positives. Considering all nodules regardless of the size and composition, MRI sensitivity was 65.3% (95% CI: 56.3–73.2) and the negative predictive value (NPV) was 43.8% (95% CI: 33.0–55.2), driven by the high proportion of small nodules missed. When stratifying by size (≥ 4 mm), sensitivity increased to 82.4% (95% CI: 72.2–89.4), with an accuracy of 86.9% (95% CI: 79.2–92.0).

### Detection performance according to composition

When stratified by composition, we observed an excellent ability of readers in identifying solid and part-solid nodules with MRI, with a sensitivity of 83.1% (95% CI: 73.2–89.9), PPV of 98.5% (95% CI: 91.8–99.7), and an accuracy of 87.3% (95% CI: 79.8–92.3).

In contrast, for calcified nodules, MRI showed fair sensitivity (28.6%; 95% CI: 16.3–45.1) and negative predictive value (NPV) (56.1% (95% CI: 43.3–68.2)) (Table 2).

**Table 2:** Diagnostic performance of lung MRI in the detection of nodules in comparison to PCD-CT according to lung nodules' size and composition

	TP	FP	FN	TN	Sensitivity	Specificity	PPV	NPV	Accuracy
Overall nodules	77	1	41	32	0.653 [0.563–0.732]	0.97 [0.847–0.995]	0.987 [0.931–0.998]	0.438 [0.33–0.552]	0.722 [0.646–0.787]
≥ 4 mm	61	1	13	32	0.824 [0.722–0.894]	0.97 [0.847–0.995]	0.984 [0.914–0.997]	0.711 [0.566–0.823]	0.869 [0.792–0.92]
< 4 mm	13	0	25	32	0.342 [0.212–0.501]	1.0 [0.893–1.0]	1.0 [0.772–1.0]	0.561 [0.433–0.682]	0.643 [0.526–0.745]
**Performance according to nodule composition**									
Solid and part-Solid nodule	64	1	13	32	0.831 [0.732–0.899]	0.97 [0.847–0.995]	0.985 [0.918–0.997]	0.711 [0.566–0.823]	0.873 [0.798–0.923]
Calcific and part-Calcific	10	0	25	32	0.286 [0.163–0.451]	1.0 [0.893–1.0]	1.0 [0.722–-1.0]	0.561 [0.433–0.682]	0.627 [0.507–0.733]
**Performance according to nodule composition and size**									
Solid < 4 mm	10	1	9	32	0.526 [0.317–0.727]	0.97 [0.847–0.995]	0.909 [0.623–0.984]	0.78 [0.633–0.88]	0.808 [0.681–0.892]
Solid ≥ 4 mm	53	1	1	32	0.981 [0.902–0.997]	0.97 [0.847–0.995]	0.981 [0.902–0.997]	0.97 [0.847–0.995]	0.977 [0.92–0.994]
Solid ≥ 6 mm	28	1	0	32	1.0 [0.879–1.0]	0.97 [0.847–0.995]	0.967 [0.823–0.994]	1.0 [0.893–1.0]	0.984 [0.913–0.997]
Solid ≥ 8 mm	15	0	0	32	1.0 [0.796–1.0]	1.0 [0.893–1.0]	1.0 [0.796–1.0]	1.0 [0.893–1.0]	1.0 [0.924–1.0]

### Detection performance in solid nodules according to size

MRI showed higher performance for nodules ≥ 4 mm, with a sensitivity of 98.1% (95% CI: 90.2–99.7), specificity of 97% (95% CI: 84.7–99.5), and an accuracy of 97.7% (95% CI: 92.0–99.4). MRI accuracy further increased with larger threshold size, reaching 98.4% (95% CI: 91.3–99.7) for nodules ≥ 6 mm and 100% [95% CI: 92.4–100] for nodules ≥ 8 mm (Table 2). Therefore, MRI performance is strongly size-dependent, showing reduced sensitivity for lesions smaller than 4 mm.

Logistic regression analysis demonstrated that both nodule composition and size influenced MRI detection. Solid nodules were over 100 times more likely to be detected than calcific ones (OR = 149.6; 95% CI: 14.7–1524.2; *p* < 0.001). Additionally, for each 1 mm increase in CT-measured diameter, the odds of MRI detection tripled (OR = 3.2; 95% CI: 1.7–6.1; *p* < 0.001), supporting a strong positive association between size, composition, and detectability.

### Inter-reader agreement

Inter-reader agreement in the detection of nodules was substantial for both PCD-CT (95% agreement, Cohen’s *K* = 0.77 [95% CI 0.38–1.00]) and MRI (90% agreement, Cohen’s *K* = 0.88 [0.70–1.00]; McNemar test not significant).

Excellent reliability in nodule sizing was observed for both PCD-CT (ICC = 0.993 [0.929–1.000], CCC = 0.993) and MRI (ICC = 0.997 [0.962–0.999], CCC = 0.997).

The mean difference of 0.10 mm; 95% LoA [−1.80 to 2.00]mm for PCD-CT and of −0.33 mm; 95% LoA [−1.64 to 0.97]mm for MRI.

## Discussion

Lung MRI is an appealing radiation-free alternative to CT for population-level screening and radiation-sensitive groups [19–21].

Our aim was to assess whether an optimized MRI sequence could achieve diagnostic performance comparable to CT—specifically in detection rate—while avoiding ionizing radiation. We employed an optimized T1w-3D-FFE sequence at 3-T MRI, acquired in a single breath-hold of approximately 17 s.

Overall, MRI demonstrated an accuracy of 87.3% for detecting solid nodules of any size, which increased to 97.7% (95% CI: 92.0–99.4) for nodules ≥ 4 mm.

MRI systematically underestimated nodule size compared to PCD-CT, with a difference of 1.02 mm (95% CI: [−1.16, 3.20] mm), resulting in the lower limit of what was previously reported (1–2 mm underestimation of MRI compared to CT) [22, 23]. This is directly caused by partial volume effects related to our 2 × 2 × 2 mm isotropic resolution, which smooths nodule edges compared to HRCT. This systematic bias may have direct clinical implications near key Lung-RADS size thresholds (6–8 mm), where a ~ 1 mm underestimation may cause nodule category migration, underscoring the need for MRI-specific size criteria rather than direct application of CT-based thresholds. However, in our cohort, underestimation did not significantly impact the detection of clinically relevant nodules, as supported by the excellent agreement on Lung-RADS classification. Notably, 3 patients were identified with suspicious lesions on MRI (Lung-RADS 4A-4B) and confirmed as cancer by CT, FDG-PET, and histology. No cases of clinically relevant nodules were missed by MRI. A few cases of disagreement between MRI and CT were reported. For smaller nodules on MRI, 2 patients with Lung-RADS 1 on MRI were reclassified as Lung-RADS 2 on CT, while for the solid appearance of a large calcific nodule on MRI, 5 patients with Lung-RADS 2 on MRI were downgraded as Lung-RADS 1 on CT.

MRI detection rate was affected by both nodule size and composition. It was unreliable for detecting small nodules < 4 mm, particularly calcified ones, representing 51.2% of undetected nodules (median size 2 mm), due to the low hydrogen proton content, resulting in signal voids on MRI [9]. 48.8% of the remaining undetected nodules were solid, but with a very small size (median diameter 2.5 mm [IQR 2.1–3.0 mm]).

Although nodules < 4 mm and calcific nodules are generally considered benign in the setting of lung cancer screening [16], small malignant nodules could be missed in oncologic patients, limiting MRI’s applicability for detecting distant pulmonary metastasis in oncological patients.

Optimization of Lung MRI balancing accuracy and acquisition time is a major issue for the clinical applicability of MRI.

Recently introduced ultrashort echo time (UTE) and zero echo time (ZTE) sequences allow good image quality, despite the lungs’ inherently short T2*, and agreement with CT [9, 24].

However, their routine use is limited by prolonged scan times (7–10 min) [25, 26], potentially affecting the evaluation of lung parenchyma near the heart [27], and in the subpleural region [25]. In contrast, Cartesian acquisitions combined with state-of-the-art gradient systems and AI-based reconstruction techniques can achieve echo times below 1 millisecond, enabling full chest acquisition within a single breath-hold, representing a more practical and scalable solution for high-throughput lung MRI.

A key technical focus of our study was the use of AI-powered CS, which mitigates the trade-off between acquisition speed and image quality compared to conventional CS. By integrating deep learning into the reconstruction process, this method improves noise–structure separation and preserves spatial detail, allowing high acceleration factors. Additionally, deep-learning-based denoising algorithms have been shown to preserve image quality and fine structural details [28].

Nevertheless, minimal smoothing and partial-volume effects may still occur, particularly for very small nodules (< 4 mm). A careful balance between speed and quality is necessary to preserve MRI sensitivity.

In fact, we limited the denoising level to 40% to limit the impact on edge sharpness and nodule boundary definition [29], potentially impacting sizing.

A previous study [14] identified CS-AI-10 as the optimal acceleration factor, offering a good compromise between scan time reduction, image quality and detection rate, with a scan time of 2:34 min. Nonetheless, free-breathing acquisitions depend on regular respiratory patterns, which cannot be guaranteed in patients with impaired lung function and are susceptible to motion artifacts. Additionally, Marka et al [30] evaluated an AI-CS 3D GRE sequence for lung nodule detection, reporting a detection rate of 96.3% for nodules > 3 mm, but with a total scan time of 3:53 min and respiratory motion artifacts contributing to missed small nodules.

Our optimized T1w-3D-FFE sequence with a CS-AI-9 allows a single breath-hold acquisition (up to 17 s), achieving rapid scanning with preserved image quality and good detection performance for clinically relevant nodules, enabling competitive comparison with a CT-based screening protocol.

Detection of solid nodules was excellent despite their small median size, comparable to previous studies on UTE and ZTE sequences [24, 25], as supported by a recent meta-analysis showing a sensitivity of 87.7% (95% CI: 81.1, 92.2) for nodules ≥ 4 mm [18].

This may reflect improved image quality and the absence of artifacts typically associated with respiratory triggering [25].

Moreover, compared to other studies on lung MRI for nodules detection, our cohort exhibited smaller nodules (4.0 mm [IQR 3.5–7.0 mm] on MRI), likely reflecting differences in patient characteristics, as our population consisted of asymptomatic healthy individuals rather than patients with a high likelihood of lung disease [22, 23, 26].

A limitation of the study is the relatively small sample size, which reflects the low prevalence of lung nodules in the general population [31], and with a small number of sub-solid nodules, thus further validation in high-risk populations is needed to confirm generalizability.

The 2 × 2 × 2 mm spatial resolution and Dixon echo time constraints limit the detection of sub-4 mm nodules, where MRI sensitivity drops to 0.342 and 0.526 for all nodules and solid nodules, respectively and contributes to size underestimation, introducing a risk of nodule category migration near screening thresholds, underscoring the need for MRI-specific size criteria. Finally, although similar AI-CS reconstruction algorithms are available from multiple vendors, differences in implementation and denoising effects may influence reproducibility across platforms, requiring multicentric and multi-vendor validation.

In conclusion, lung MRI represents a promising alternative to CT for lung nodule screening, with potential applicability both for population-wide lung cancer screening and selected subgroups such as younger individuals, patients requiring repeated follow-up examinations or those who decline CT.

It offers the advantage of radiation-free detection and longitudinal monitoring, along with the potential of integration with other MRI sequences such as STIR [27] and DWI [32], which can further enhance nodule characterization in high-risk populations. Furthermore, recent advances in low-field-strength closed-bore MRI technology may reduce scanning costs while minimizing susceptibility artifacts at air-tissue interfaces [33].

## Abbreviations

AI-CS: Artificial intelligence-assisted compressed sensing
CCC: Lin’s Concordance Correlation Coefficient
FFE: Fast field echo
MRI: Magnetic resonance imaging
PCD-CT: Photon-counting detector-computed tomography
